# Navigating Challenges and Advances in Pediatric Psoriasis and Atopic Dermatitis Treatment

**DOI:** 10.3390/children10121909

**Published:** 2023-12-11

**Authors:** Katie K. Lovell, Lindsay C. Strowd, Steven R. Feldman

**Affiliations:** 1 Department of Dermatology, Center for Dermatology Research, Wake Forest University School of Medicine, Winston-Salem, NC 27104, USA; lchaney@wakehealth.edu (L.C.S.); sfeldman@wakehealth.edu (S.R.F.); 2 Department of Dermatology, Wake Forest University School of Medicine, Winston-Salem, NC 27104, USA; 3 Department of Pathology, Wake Forest University School of Medicine, Winston-Salem, NC 27104, USA; 4 Department of Social Sciences & Health Policy, Wake Forest University School of Medicine, Winston-Salem, NC 27104, USA

The contents of this Special Issue provide a broad overview of the current landscape of psoriasis and atopic dermatitis (AD) treatment in pediatric populations, highlighting the challenges and recent advances. Despite affecting approximately 1% of children [1], options for pediatric psoriasis remain limited despite the growing number of treatments available for adults. On the other hand, AD is one of the most prevalent systemic inflammatory diseases in childhood, with a multitude of therapeutics in the pipeline [2]. As discussed in Chovatiya and Silverberg’s review of the pathophysiology of both diseases, the enhanced insight into the disease mechanisms has led to a “therapeutic revolution”. Recent and coming advancements continue to provide hope for an era of personalized medicine in the management of pediatric psoriasis and AD. This Special Issue features six articles covering a range of topics on the management of pediatric psoriasis and atopic dermatitis. Since four years have elapsed since the publishing of these articles, this editorial serves to briefly summarize the contents of the Special Issue and provide pertinent updates.

Approximately one-third of psoriasis cases begin in the pediatric years [1]. Approved psoriasis medications in children are limited when compared to adults. Frantz et al. provided a broad overview of the topicals available for the treatment of pediatric AD (contribution 1). In addition to these topicals, new psoriasis topicals like roflumilast and tapinarof have been approved or have ongoing clinical trials in the pediatric population. Both medications are steroid-sparing agents with limited systemic absorption, increasing their favorability for use in the pediatric population [3]. Roflumilast is a phosphodiesterase-4 (PDE-4) inhibitor that is approved in children as young as age 6. In two phase 3 studies (DERMIS-1 and -2), more subjects in the roflumilast group had Investigator Global Assessment (IGA) success than the vehicle (~40% vs. ~6%, *p* < 0.001) [3]. Most patients tolerated the medication well, with a <2% discontinuation rate with adverse effects of stinging and burning at the application site, similar to the vehicle [3].

Tapinarof is an aryl hydrocarbon receptor modulator with an ongoing clinical trial in the pediatric population (NCT05172726). With 35–40% of patients achieving Investigator Global Assessment (IGA) success compared to 6.0–6.3% of the control group (*p* < 0.001) in two phase 3 studies (PSOARING 1 and 2), tapinarof is also an efficacious option (contribution 1). The most common adverse event was folliculitis, which was observed in 20% of the tapinarof group versus 1% of the control group [3].

For pediatric patients with mild AD, roflumilast and tapinarof have also been studied. A phase 3 trial studying the efficacy of roflumilast for atopic dermatitis in ages 2 years and up was recently completed in July of 2023 (NCT04845620). No results have been posted for this study, but a supplemental new drug application was submitted in the fall of 2023 for the use of roflumilast in AD.

Tapinarof is approved for patients with AD (ages 12 and up), with a phase 3 study evaluating its safety and efficacy in individuals as young as age 2. About 50% of tapinarof patients reached an IGA of 0-1 compared to 24% of vehicle patients, with treatment success maintained for 4 weeks after the study’s completion [4].

Topical ruxolitinib is a Janus kinase (JAK) 1/2 inhibitor which was approved in 2021 for AD use in ages 12 and up [4]. This medication bolsters a rapid reduction in itch, with about 50% of patients achieving a 4-point reduction in itching [4]. However, like oral JAK inhibitors, ruxolitinib carries black box warnings, which, even if not clinically meaningful, may be alarming to parents.

How helpful these new topical treatments are will depend on how well patients use them. Adherence to topical treatment, even in children suffering from skin diseases, can be abysmal. Poor adherence to topical treatment may be a major hurdle to advancing care for psoriasis and atopic dermatitis in children.

In severe pediatric psoriasis, biologics have superior efficacy in decreasing psoriasis severity scores when compared to other systemic agents, like methotrexate [5]. Currently, five biologic agents are approved for use in pediatric psoriasis patients: etanercept; adalimumab; ustekinumab; and, since Cline et al., new additions of ixekizumab and secukinumab (contribution 2). The accessibility and practicality of these biologics in the pediatric population may lead to limitations. The costliness of biologic agents, in contrast to more economic options like methotrexate, may result in biologics being inaccessible until other options have been exhausted [5]. Despite the invasive route of administration, biologics are associated with a better drug survival rate (rate and duration of adherence to a drug) over methotrexate, which requires routine lab monitoring [5]. While an injectable may be less preferable than an oral option, the safety of available oral alternatives approved for use in children is limited. Ideally, the future development of medications for this population will yield a product that is well-balanced between safety and efficacy, with options for oral administration.

The introduction of dupilumab several years ago was a revolutionary advancement in atopic dermatitis treatment. With approval extending down to 6 months of age, the medication boasts both high efficacy and excellent safety, and is supported by nearly 5 years of safety data [2]. Some considerations regarding the use of biologics in the pediatric population, especially in infancy and the preschool years, include the management of live vaccines, the route of administration, and the requirement for long-term regular use of the medication. The risk of conjunctivitis associated with dupilumab may limit its use in affected patients, although the conjunctivitis can be managed [2]. Biologics like tralokinumab and lebrikizumab, which bind and inhibit interleukin-13 (IL-13), may have less risk of conjunctivitis and are currently under study for patients as young as 12 years old [6].

An alternative target of interest in AD management is IL-31, which is promising for patients whose itching does not respond to IL-4 or IL-13 blockade. However, IL-31 inhibition does not address AD-associated inflammation as effectively as other targets and may necessitate the use of combination therapy [6]. More data on the chronic use of these medications and potential tapering regimens are crucial for refining pediatric AD management strategies.

The phase 3 trial of the oral JAK1/2 inhibitor upadacitinib was discussed in a contribution by Frantz et al. (contribution 1). Upadacitinib was approved in January of 2022 and is now available for the management of atopic dermatitis in children aged 12 and older. While JAKs provide very rapid itch relief, they come with a higher incidence of adverse events and necessitate regular lab monitoring [6]. Some experts propose that JAK inhibitors could play a role in episodic AD management rather than the long-term approach seen with biologics [6]. Additionally, for patients with concomitant alopecia areata or vitiligo, JAK inhibitors are an excellent option for the management of concomitant disease processes [6]. As Frantz et al. alluded, the long-term safety of oral JAK inhibitors will need to be further characterized and, until then, may continue to be limited in clinical practice (contribution 1).

Regardless of the advances in pharmacologic options for AD, many patients and their families seek alternative therapies for control of their AD, making complementary and integrative therapies a growing area of study. In this special edition, Adler-Neal et al. discuss multiple randomized, controlled clinical trials related to probiotics, vitamins, and Chinese herbal treatments for pediatric AD (contribution 3). The research identifies promising outcomes, particularly with certain strains of probiotics like *L. plantarum* and *L. fermentum*, showcasing improvements in clinical severity scores. However, further research is warranted to offer patients safe and efficacious non-pharmacologic alternatives.

In addition, Lucas et al. discussed a possible a new lotion, Dermacare, which improved skin moisturization, reduced transepidermal water loss (TEWL), and positively impacted objective and subjective parameters in pediatric AD patients (contribution 4). These results suggest that Dermacare, with regular use, could potentially be used as a complementary topical product to reduce the risk of relapse and extend steroid-free treatment periods in AD patients. However, further studies are recommended in order to confirm these findings and explore the potential benefits of prolonged use.

In exploring the complexities of managing psoriasis and atopic dermatitis in the pediatric population, this Special Issue addresses many of the recent developments within the field. As Na et al. emphasize, pediatric psoriasis and AD can have a profound impact on a child’s quality of life, and their management warrants a multidisciplinary approach to care, including educational and psychological support (contribution 5). Despite the promising results from clinical trial data, the efficacy of the new topicals in psoriasis and atopic dermatitis must contend with the pervasive problem of adherence seen with topical use in dermatology. In severe cases of these diseases, the expansion of systemic therapies also introduces challenges. The decreased specificity of some targets may lead to increased side effects, and the high cost of biologics, though efficacious, could restrict accessibility. The quest for a personalized, well-balanced treatment incorporating safety, efficacy, and accessibility remains paramount, especially within the pediatric population.

## Data Availability

No new data were created or analyzed in this study. Data sharing is not applicable to this article.
